# Minimum Information about T Regulatory Cells: A Step toward Reproducibility and Standardization

**DOI:** 10.3389/fimmu.2017.01844

**Published:** 2018-01-15

**Authors:** Anke Fuchs, Mateusz Gliwiński, Nathali Grageda, Rachel Spiering, Abul K. Abbas, Silke Appel, Rosa Bacchetta, Manuela Battaglia, David Berglund, Bruce Blazar, Jeffrey A. Bluestone, Martin Bornhäuser, Anja ten Brinke, Todd M. Brusko, Nathalie Cools, Maria Cristina Cuturi, Edward Geissler, Nick Giannoukakis, Karolina Gołab, David A. Hafler, S. Marieke van Ham, Joanna Hester, Keli Hippen, Mauro Di Ianni, Natasa Ilic, John Isaacs, Fadi Issa, Dorota Iwaszkiewicz-Grześ, Elmar Jaeckel, Irma Joosten, David Klatzmann, Hans Koenen, Cees van Kooten, Olle Korsgren, Karsten Kretschmer, Megan Levings, Natalia Maria Marek-Trzonkowska, Marc Martinez-Llordella, Djordje Miljkovic, Kingston H.G. Mills, Joana P. Miranda, Ciriaco A. Piccirillo, Amy L. Putnam, Thomas Ritter, Maria Grazia Roncarolo, Shimon Sakaguchi, Silvia Sánchez-Ramón, Birgit Sawitzki, Ljiljana Sofronic-Milosavljevic, Megan Sykes, Qizhi Tang, Marta Vives-Pi, Herman Waldmann, Piotr Witkowski, Kathryn J. Wood, Silvia Gregori, Catharien M. U. Hilkens, Giovanna Lombardi, Phillip Lord, Eva M. Martinez-Caceres, Piotr Trzonkowski

**Affiliations:** ^1^GMP facility, DFG-Center for Regenerative Therapies Dresden (CRTD), Center for Molecular and Cellular Bioengineering (CMCB), and Department of Internal Medicine I, University Hospital Carl Gustav Carus, Technische Universität Dresden, Dresden, Germany; ^2^Department of Clinical Immunology and Transplantology, Medical University of Gdańsk, Gdańsk, Poland; ^3^MRC Centre for Transplantation, King’s College London, Guy’s Hospital, London, United Kingdom; ^4^Institute of Cellular Medicine, Newcastle University, Newcastle upon Tyne, United Kingdom; ^5^Department of Pathology, University of California, San Francisco, San Francisco, CA, United States; ^6^Broegelmann Research Laboratory, Department of Clinical Science, University of Bergen, Bergen, Norway; ^7^Pediatric Stem Cell Transplantation and Regenerative Medicine, Department of Pediatrics, Stanford School of Medicine, Stanford, CA, United States; ^8^Diabetes Research Institute, IRCCS San Raffaele Scientific Institute, and TrialNet Clinical Center, San Raffaele Hospital, Milan, Italy; ^9^Department of Immunology, Genetics and Pathology, Uppsala University, Uppsala, Sweden; ^10^Department of Pediatrics, Division of Blood and Marrow Transplantation, University of Minnesota, Minnesota, MN, United States; ^11^Hormone Research Institute, University of California, San Francisco, San Francisco, CA, United States; ^12^Department of Immunopathology, Sanquin Research and Landsteiner Laboratory, University of Amsterdam, Academic Medical Center, Amsterdam, Netherlands; ^13^Department of Pathology, Immunology, and Laboratory Medicine, University of Florida Diabetes Institute, College of Medicine, Gainesville, FL, United States; ^14^Laboratory of Experimental Hematology, Vaccine & Infectious Disease Institute, Faculty of Medicine and Health Sciences, University of Antwerp, Antwerp University Hospital (UZA), Edegem, Belgium; ^15^Centre de Recherche en Transplantation et Immunologie UMR1064, INSERM, Université de Nantes, Nantes, France; ^16^Division of Experimental Surgery, Department of Surgery, University Hospital Regensburg, Regensburg, Germany; ^17^Allegheny Health Network, Institute of Cellular Therapeutics, Carnegie Mellon University, Pittsburgh, PA, United States; ^18^Transplant Institute, Department of Surgery, The University of Chicago, Chicago, IL, United States; ^19^Departments of Neurology and Immunobiology, Yale School of Medicine, New Haven, CT, United States; ^20^Nuffield Department of Surgical Sciences, John Radcliffe Hospital, University of Oxford, Oxford, United Kingdom; ^21^Department of Medicine and Aging Sciences, University of Chieti-Pescara, Chieti, Italy; ^22^Department for Immunology and Immunoparasitology, National Reference Laboratory for Trichinellosis, Institute for the Application of Nuclear Energy, University of Belgrade, Belgrade, Serbia; ^23^National Institute for Health Research Newcastle Biomedical Research Centre at Newcastle upon Tyne Hospitals NHS Foundation Trust and Newcastle University, Newcastle upon Tyne, United Kingdom; ^24^Department of Gastroenterology, Hepatology, Endocrinology, Diabetology, Transplantationsforschungszentrum, Medical School of Hannover (MHH), Hannover, Germany; ^25^Laboratory of Medical Immunology, Department of Laboratory Medicine, Radboudumc, Nijmegen, Netherlands; ^26^Immunology-Immunopathology-Immunotherapy (i3), UPMC Univ Paris 06, UMRS 959, Sorbonne Université, and Biotherapy (CIC-BTi) and Inflammation-Immunopathology-Biotherapy Department, AP-HP, Hôpital Pitié-Salpêtrière, Paris, France; ^27^Department of Nephrology, Leiden University Medical Center, Leiden, Netherlands; ^28^Department of Immunology, Genetics and Pathology, Rudbeck Laboratory, Uppsala University Hospital, Uppsala, Sweden; ^29^Transplantation Immunology, Gothenburg University, Gothenburg, Sweden; ^30^Molecular and Cellular Immunology/Immune Regulation, DFG-Center for Regenerative Therapies Dresden (CRTD), Center for Molecular and Cellular Bioengineering (CMCB), Technische Universität Dresden, and Paul Langerhans Institute Dresden (PLID) of the Helmholtz Zentrum München at the University Hospital and Medical Faculty Carl Gustav Carus of TU Dresden, Dresden, Germany; ^31^German Center for Diabetes Research (DZD e.V.), Neuherberg, Germany; ^32^Department of Surgery, Faculty of Medicine, The University of British Columbia, BC Children’s Hospital Research Institute, Vancouver, BC, Canada; ^33^Laboratory of Immunoregulation and Cellular Therapies, Department of Family Medicine, Medical University of Gdańsk, Gdańsk, Poland; ^34^Medical Research Council Centre for Transplantation, Institute of Liver Studies, King’s College London, London, United Kingdom; ^35^Department of Immunology, IBISS, University of Belgrade, Belgrade, Serbia; ^36^Immune Regulation Research Group, School of Biochemistry and Immunology, Trinity Biomedical Sciences Institute, Trinity College Dublin, Dublin, Ireland; ^37^Faculty of Pharmacy, Research Institute for Medicines (iMed.ULisboa), Universidade de Lisboa, Lisbon, Portugal; ^38^Departments of Microbiology & Immunology and Medicine, Faculty of Medicine, McGill University, Program in Infectious Disease and Immunity in Global Health, Centre of Excellence in Translational Immunology (CETI), Research Institute of McGill University Health Centre, Montréal, QC, Canada; ^39^College of Medicine, Nursing and Health Sciences, Regenerative Medicine Institute (REMEDI), Biomedical Sciences, National University of Ireland, Galway, Ireland; ^40^Division of Stem Cell Transplantation and Regenerative Medicine, Department of Pediatrics, ISCBRM, Stanford School of Medicine, Stanford, CA, United States; ^41^WPI Immunology Frontier Research Center, Osaka University, Osaka, Japan; ^42^Department of Clinical Immunology, Hospital Clínico San Carlos, Universidad Complutense of Madrid, Madrid, Spain; ^43^Institute for Medical Immunology, Charité – Universitätsmedizin Berlin, Corporate Member of Freie Universität Berlin, Humboldt-Universität zu Berlin and Berlin Institute of Health, Berlin, Germany; ^44^Columbia Center for Translational Immunology, Columbia University College of Physicians and Surgeons, Bone Marrow Transplantation Research, Division of Hematology/Oncology, Columbia University Medical Center, Columbia University, New York, NY, United States; ^45^Department of Surgery, University of California, San Francisco, San Francisco, CA, United States; ^46^Immunology of Diabetes Unit, Germans Trias i Pujol Research Institute (IGTP), Barcelona, Spain; ^47^Sir William Dunn School of Pathology, University of Oxford, Oxford, United Kingdom; ^48^Mechanisms of Peripheral Tolerance Group, San Raffaele Telethon Institute for Gene Therapy (SR-TIGET), San Raffaele Scientific Institute IRCCS, Milan, Italy; ^49^School of Computing, Newcastle University, Newcastle upon Tyne, United Kingdom; ^50^Immunology Division, Germans Trias i Pujol University Hospital - Can Ruti, Department Cellular Biology, Physiology, Immunology, Universitat Autònoma Barcelona, Badalona, Spain

**Keywords:** minimum information model, T regulatory cells, immunotherapy, good manufacturing practice, cell therapy, immune tolerance

## Abstract

Cellular therapies with CD4+ T regulatory cells (Tregs) hold promise of efficacious treatment for the variety of autoimmune and allergic diseases as well as posttransplant complications. Nevertheless, current manufacturing of Tregs as a cellular medicinal product varies between different laboratories, which in turn hampers precise comparisons of the results between the studies performed. While the number of clinical trials testing Tregs is already substantial, it seems to be crucial to provide some standardized characteristics of Treg products in order to minimize the problem. We have previously developed reporting guidelines called minimum information about tolerogenic antigen-presenting cells, which allows the comparison between different preparations of tolerance-inducing antigen-presenting cells. Having this experience, here we describe another minimum information about Tregs (MITREG). It is important to note that MITREG does not dictate how investigators should generate or characterize Tregs, but it does require investigators to report their Treg data in a consistent and transparent manner. We hope this will, therefore, be a useful tool facilitating standardized reporting on the manufacturing of Tregs, either for research purposes or for clinical application. This way MITREG might also be an important step toward more standardized and reproducible testing of the Tregs preparations in clinical applications.

## Introduction

T regulatory cells (Tregs) are dominant cellular compounds of the immune system protecting the body from autoimmune reactions. These cells are also involved in imposing tolerance to alloantigens such as transplanted allogeneic cells and tissues (1–5). For all these reasons, several Treg-based therapeutics are being tested in clinical trials as a prophylaxis or treatment of autoimmune diseases, graft-versus-host disease after hematopoietic stem cell transplants or rejections after solid organ transplants (6). The list of potential applications in the future is even wider. At the same time, manufacturing of Tregs for preclinical and clinical experiments varies considerably between different centers, which significantly diminishes possible comparisons between the trials. For this reason, future development of these therapies is hampered as it happens that the available results from different trials are contradictive. The specificity of cellular products makes it difficult to verify the results in huge multicentre trials and therefore better standardization of early-phase trials as well as cellular products themselves might facilitate the progress in this promising branch of medicine.

We propose here a tool for standardization of Tregs studies designed on the basis of so-called minimum information models (MIMs). These models have gained increasing popularity among scientists as they enable the interpretation of reported data, comparison between data from different studies and facilitate experimental reproducibility (7, 8). MIMs provide mechanisms that all laboratories report at least the key facts about their analysis in a clear and consistent manner, allowing a comparison across the whole field. Our consortium has already designed the MIM called minimum information about tolerogenic antigen-presenting cells (MITAP). This is a reporting framework that makes transparent differences and similarities of different tolerogenic antigen-presenting cells (tolAPC) (9). It provides minimum reporting guidelines for the production process of tolAPC used in preclinical and/or clinical studies. We have followed the MITAP experience and designed a MIM for the manufacture of Tregs. We call it minimum information about T regulatory cells (MITREG). MITREG will be a useful resource for investigators reporting their data on the use of *in vitro* expanded natural Tregs or induced Tregs in preclinical models or clinical trials.

## Methods

### Setting Up MITREG: Community Building and Initial Analysis

The community was mainly built on the experience of our completed MITAP initiative. For several years now, we have been working together in the field of tolerogenic cellular therapies under the umbrella of the consortium AFACTT (action to focus and accelerate cell-based tolerance-inducing therapies—http://www.afactt.eu/). It brings together European scientists and clinicians with the aim of jointly addressing issues related to the translation and clinical application of these new treatments. Having the experience of MITAP, we used this document as a template to describe Treg therapies. For MITREG, we also tried to extend the initiative beyond Europe and invited scientists working on tolerogenic cellular therapies from around the world. This way we ensured a broadly reflective discussion taking into account various opinions and current practices of many laboratories within the discipline.

The work on this MITREG document covered a series of “exercises” that provided some initial data. Like for MITAP, the exercises aimed at gathering “terms” in order to acquire basic vocabulary in use within the community. The first, so-called “sticky-note” exercise performed at several AFACTT meetings assumed that each participant wrote a term on a sticky-note; these were then collated and clustered on a wall by the whole group, identifying synonyms and related terms. Second, we used the MITAP template to incorporate the collected terms and created an initial version of MITREG. This document underwent several rounds of face-to-face and online consultations with AFACTT members to improve its clarity. Internally agreed version was circulated to external specialists in the field. This external feedback was collected and implemented in the final version of the MITREG document. Finally, we used the existing literature to obtain a picture of how well the required information has been described in published articles.

## Results

### Overview of the MITREG Document

The design of the MITREG document followed the concept of MITAP, which facilitated the whole process. It describes the manufacturing of Treg products in a chronological way. The document is divided into four sections highlighting critical points of the process and regulatory issues. The document describes the details that should be provided by investigators, which would allow other researchers to repeat the process. It also advises on the use of existing taxonomies and databases to provide the information in a uniform manner, and it suggests the use of other MIMs where appropriate. The full MITREG document can be found on archive.org (http://w3id.org/ontolink/mitreg) and it is also included in the Appendix A (MITREG document).

### Section 1: Cells at the Start of the Procedure

This section describes characteristics of the biological material *before* it undergoes any manipulation. There are five subparts asking for (a) essential information about the donor, (b) source of the cells, (c) the methods used to separate Tregs, (d) the phenotype after separation, and (e) the number of Tregs after separation.

### Section 2: Expansion/Differentiation

This section describes the protocol that has been used to expand or differentiate Tregs. The specificity of Tregs was a challenge here as different subsets can be obtained with a wide range of methods. Tregs can be either isolated and optionally expanded or can be induced from naive precursors. There are five subsections giving details on (a) preculture conditions, (b) culture conditions, (c) the protocol used to expand or differentiate cultured Tregs, (d) stimuli used during the process, and (e) the way Tregs are stored immediately after expansion/differentiation.

### Section 3: Cells after Expansion/Differentiation

This section describes the characteristics of Tregs *after* the expansion or differentiation. It is mainly focused on the phenotype of the final Treg product as well as its suppressive activity verified in any form of functional assay. It also documents the cell yield from the entire process and, if the product is for clinical use or testing of adoptive transfer in animals, the details on administration of the cells to the recipient.

### Section 4: About the Protocol

This final section describes remaining details of the experimental or clinical protocol such as primary or secondary goals as well as regulatory issues such as adherence to particular acts or directives including compliance with good practice requirements (GCP, GLP, or GMP guidelines). Finally, the name and contact details of the corresponding author(s) must be provided.

The MITREG document is accompanied by a handy checklist to assist investigators in ensuring that all the relevant detail is provided before submitting their manuscripts for publication. The checklist can be found at archive.org (http://w3id.org/ontolink/mitreg) and is also included in the Appendix B (MITREG checklist).

### Prevalence of MITREG Data in Extant Published Articles

The purpose of the MITREG document is to ensure that authors provide sufficient basic information about their production protocol. An implicit assumption is that currently some or all of this information is not being routinely described. To test this assumption, we reviewed a number of articles about Treg products and for each we determined whether it included data described in the MITREG document.

**Figure 1:**
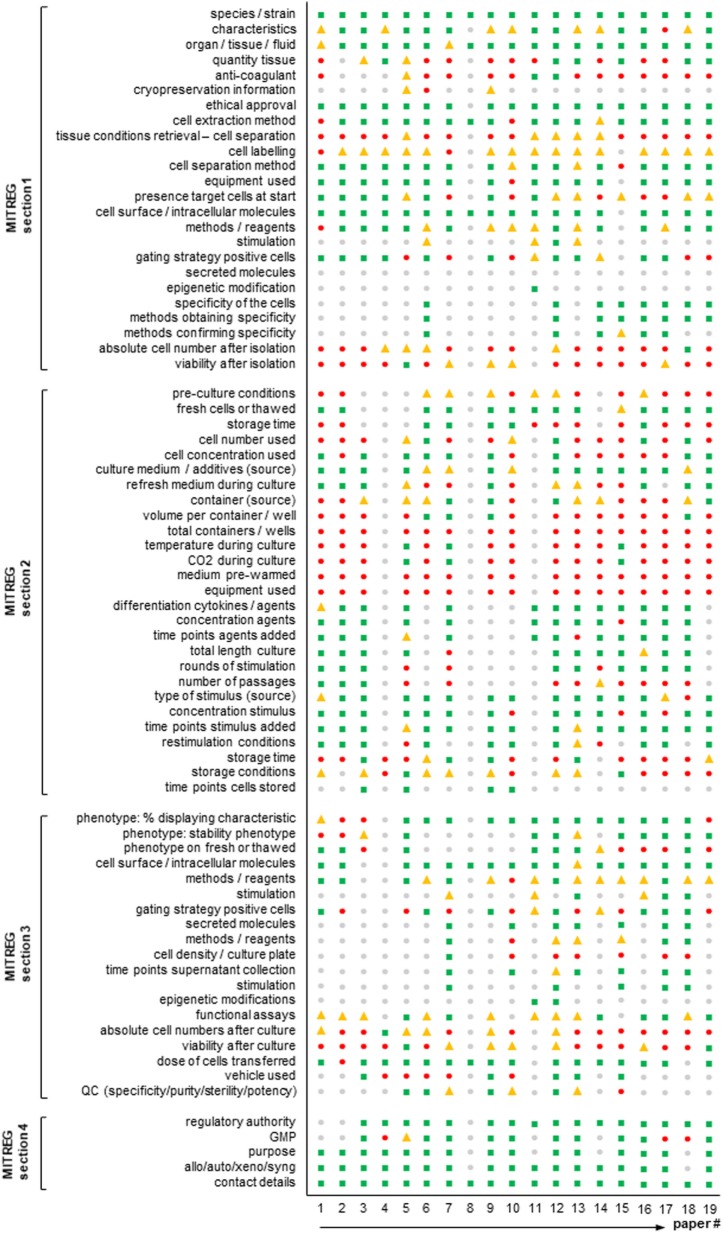
Agreement of published T regulatory cell (Treg) articles with the minimum information about T regulatory cell (MITREG) document. Graph showing the results of a total of 19 Treg articles (10–28). The order in the figure is anonymized and different from that in the references. MITREG data directly stated in the article (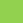 green squares), partly stated in the article (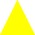 yellow triangles), not present at all (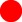 red circles), or not present as it was not relevant for the publication (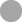 gray circles).

In detail, 19 Treg articles were selected (predominantly from members of AFACTT or from researchers well known in the field) and read in detail. The articles are given chronologically in the references but the order in Figure 1 is different and anonymized (10–28). For each section of MITREG, we determined whether the information required was directly stated in the article (or referenced) (Figure 1: green squares), partly stated in the article (Figure 1: yellow triangles), not present at all (Figure 1: red circles), or whether information was not present due to lack of relevance for the publication (Figure 1: gray circles). For example, section 1-ai of MITREG describes the species used in the experimental setup. An article with the phrase “human” or “*Homo sapiens*” would fall into the first category (*included in the publication*). However, when mice are used and only the species is mentioned: “mouse” or “*Mus musculus*,” but not the strain, it would fall into the second category (*included but details missing*). Many articles do not describe their experimental methodology, but instead refer to another article (“as described previously”); in this case, we checked the article up to two references deep and if found, the information was considered as “present” (Figure 1: green squares), if not it was considered as “not present” (Figure 1: red circles). This work was performed by four independent scientists with experience in the field.

Results are shown in Figure 1. This figure shows that in some sections like the species, characteristics, ethics, and cell dose transferred sections, reporting is good with almost all revised articles describing these. However, other sections are often very poorly reported. For example, storage of cells, anticoagulant used and the number/viability of cells after each separate step are not described in most articles. Moreover, important information (container type, concentration of cells) to repeat the performed experiments is missing in almost all articles.

### Sustainability

We have taken particular care to consider the issues of digital sustainability for MITREG. A well-known problem with resources linked with URLs given in articles is that URLs are often lost over time: around a 25% loss 3 years after publication (29). We have, therefore, specifically addressed this issue by use of a stable identifier space; the MITREG document and checklist are hosted by archive.org, an organization committed to long-term digital preservation. In addition, we have used a permanent identifier (http://w3id.org/ontolink/mitreg) thereby providing a redirection-step.

Resources are available in a number of formats: both PDF and Word for manipulability, but also a simple HTML representation, ensuring vendor-neutrality and future-proofing, in so far as this is possible.

## Discussion

Minimum information models aid investigators by providing a specific guideline of what is required to interpret and compare experimental findings. Furthermore, reporting guidelines will facilitate independent validation of published results, a fundamental precept of scientific research. This is to our knowledge the first proposal of a minimum information standard on the description of experimental as well as clinical manufacturing and application of Tregs. The generation of MITREG was initiated by members of the European AFACTT consortium to fill a recognized gap in data reporting standards in the Treg community. MITREG was realized with the help of key international players in the Treg field.

Nine years after the first-in-man report, there are currently close to 30 recruiting or ongoing clinical trials administering Tregs in autoimmune settings, inflammatory diseases, transplantation and graft-versus-host disease (6). Clinical grade reagents for Treg isolation by magnetic activated cell sorting have become available to the growing community and off the shelf products and GMP-compatible fluorescence-based cell sorting is currently been available from multiple manufacturers of novel closed system devices, further increasing the diversity of isolation techniques (30). Given the low frequency of Tregs in the periphery, most clinical applications require an *in vitro* cell expansion culturing step classifying them as advanced therapy medicinal products. A growing number of culturing methods are being developed and published aiming at Treg induction, enhanced *ex vivo* expansion, alloreactivity and more recently, the implementation of specific T cell receptors or chimeric antigen receptors (17, 18, 25, 31–39). We are thus at a point where protocol diversity is growing exponentially, emphasizing the necessity to harmonize reporting regimens as a prerequisite of reproducibility and quality assurance. By analyzing extant articles according to the MITREG document (Figure 1), it also becomes clear that there is a big gap in what is currently being reported and what the community considers important and wants to receive in a Treg production/expansion protocol. For example, storage conditions, cell numbers and viability and anticoagulant used are almost never reported, but are most likely measured or known by the researcher. Moreover, essential information to allow experiments to be repeated is often missing.

Together with MITREG we provide a checklist that was designed with maximal flexibility to incorporate newly developed methodologies. While MITREG does not aim at uniform protocols or dictating quality checks, it is expected to enable a mere description of the growing diversity in production procedures. We expect it to mature as novel technologies arise and become a consensus guideline within the Treg community. Only by exact reporting we will be able to identify differences in Treg preparations that may help to understand results from clinical studies. We anticipate that MITREG will be a starting point for further joint efforts of the Treg community that will ultimately lead to optimized cellular therapy.

## Author Contributions

As described in the Section “Methods,” these recommendations are the common effort of all the authors, who were involved in the design, acquisition, and interpretation of available data on Tregs as well as revised critically and approved final version of the MITREG document. In addition, AF, MG, NG, and RS were involved in collecting and analysis of the data sent by the contributors and SG, CH, GL, PL, EC, and PT supervised the work and edited the article.

## Conflict of Interest Statement

The authors declare that the research was conducted in the absence of any commercial or financial relationships that could be construed as a potential conflict of interest. The reviewer LH declared a shared affiliation, with no collaboration, with several of the authors RS, JI, CH, and PL to the handling editor.

## Appendix A

### Minimum Information for T Regulatory Cells (MITREG)

#### Introduction

The purpose of this document is to enable the description of the generation of T regulatory cell (Treg) products for therapeutic application or experimental usage. It was designed to suit reports using endogenous, induced, antigen-specific, and polyclonal freshly isolated and expanded Tregs.

This document is split into four sections, each describing a different aspect of the process. Not all sections will be relevant to all Treg products.

Information in some sections of this document may be covered by other Minimum Information documents, or defined vocabularies. For example, flow cytometry is described in MIFlowCyt,^1^ microarray data by MIAME,^2^ T-cell assays by MIATA,^3^ and production of standardized tolerogenic antigen-presenting cells by MITAP,^4^ Authors are encouraged to use these resources as appropriate.

#### Use of Terminology

The key words “**must**,” “**should**,” and “**may**” in this document are to be interpreted as follows:

**must:** this word means that the information is an absolute requirement. Failure to provide this information is in strict violation of the specification.

*EXAMPLE: the species and the source of the cell material are required for all experiments*.

**should:** this word means that there may exist valid reasons for particular protocols to not provide these data, but that these data need to be provided if it is relevant to the protocol.

*EXAMPLE: if the Tregs were generated or enriched using an antigen then this must be described, although there may be protocols where polyclonal Tregs are applied*.

**may:** this word means that the data are optional and do not need to be included, but can be provided.

*EXAMPLE: the health or age of the organism can be provided, but there may be protocols where this is not assessed, even though it could be*.

These definitions are modified from RFC 2119 (https://tools.ietf.org/html/rfc2119).

**(1) Cells at the start of procedure**

This section describes the characteristics and state of the cells used in the procedure prior to any form of cell manipulation processes such as cell expansion and/or differentiation.

**(a) Essential information about the donor**

***(i) Species and strain***

The taxonomy of the organism from which the cells originated. You **must** use names according to the NCBI Taxonomy.^5^ If the strain of the species is known, you **should** indicate this.

*EXAMPLE: Homo sapiens/human; Mus musculus, Rag^−/−^*γ*_c_^−^ (B6, H-2b)*

***(ii) Characteristics of the organism***

Include information about the organism from which the cells originated that is not adequately described by the species/strain information. This **may** include details of their health, age, sex, or any treatments or environmental conditions to which they have been exposed to (e.g., medication). You **may** also include information that is specific to your laboratory, such as an individual identifier number. If you have purchased experimental animals (e.g., BALB/c mice) or tissues (e.g., human bone marrow) you **should** indicate the source of purchase.

EXAMPLE: healthy/volunteer/male/6-weeks-old/male/BALB/c mice/purchased from Charles River (Margate England)

**(b) Source of cell material**

The organ, tissue, or fluid from which the cells have been isolated **must** be stated. If you use a blood product you **should** state the product and the source (e.g., hospital department, blood bank) from where it was obtained. You **should** use terminology from Uberon,^6^ or the Foundational Model of Anatomy.^7^ You **should** also indicate the quantity of the sample by mass or volume, and, if applicable, which anti-coagulant was used. Additional details **must** be included if the source material was derived from cryopreserved samples (e.g., umbilical cord blood). This would include the methods and duration of storage and initial cell counts. The statement on use/ethics committee approval/written informed consent **MUST** be included.

*EXAMPLE: apheresis/buffy coat/bone marrow aspirate/peripheral blood, Sanquin blood supply; 250* *ml; EDTA*

**(c) Cell separation process**

***(i) Cell handling and labeling***

The methodology used to extract the cells from the source material **must** be stated. You **should** also indicate the time between cell material retrieval and start of the isolation process. You **should** indicate how the tissue was kept during this time, including the temperature and you **may** indicate the container and fluid. You **must** indicate cell labeling procedures, including characteristics and source of labeling buffers and reagents. Other details, such as cell suspension volume and concentration, incubation temperature and washing steps **should** be included.

*EXAMPLE: apheresis products were stored overnight at 4°C; Tregs were enriched by magnetic-activated cell sorting (MACS^®^ Technology); Cells were labeled with anti-CD8-coated magnetic beads (CliniMACS^®^ CD8 Reagent, Miltenyi Biotec) in 95* *ml of PBS containing 1* *mmol/l EDTA and 0.5% human albumin (PBS/EDTA buffer, Miltenyi Biotec) for 30* *min at room temperature on an orbital shaker*.

***(ii) Cell separation equipment and process***

The equipment (e.g., AutoMACS^®^, CliniMACS^®^, Aria III™ Fluorescence Activated Cell Sorter) and process used to enrich for the cells of interest **should** be stated. The presence of the target population in the starting material should be described.

*EXAMPLE: anti-CD8 bead-labeled cells were resuspended in 100* *ml of PBS/EDTA/0.5% HA. CD8^+^ cells were depleted with the use of the 2.1 depletion program on the CliniMACS^®^ Cell Separation Device (Miltenyi Biotec)*.

**(d) Phenotype**

Characteristics of the cells that have been isolated **should** be described and how this has been determined. Where only a proportion of cells in the population display a characteristic, you **should** indicate the percentage.

***(i) Cell surface and intracellular markers***

Identifying molecules that are, or are not, expressed by the cells on their surface or intracellularly is useful. You **should** describe: (1) what you measured, (2) the methodology used for the measurement (including information on reagents; if using mAbs, information on clonotype, conjugate, and manufacturer **must** be provided), (3) whether the cells received a stimulus and for how long before the measurement was carried out, and (4) the method used to set marker or population positivity (e.g., fluorescence minus one method). You **should** use cluster of differentiation (CD) names when available (e.g., use CD62L instead of the alternative name L-selectin)—a full list of regularly updated CD numbers can be found on the website run by the HCDM^8^ (human cell differentiation molecules). Otherwise, you **may** use databases, e.g., Uniprot^9^ for proteins and ChEBI^10^ for non-protein organic molecules.

*EXAMPLE: FOXP3 (PE-Cy7, clone PCH101, eBioscience) expression was measured directly after cell isolation by intracellular staining using the Foxp3/Transcription Factor Staining Buffer Set from eBioscience. Percentage of CD4^+^CD25^high^CD127^−/low^FOXP3^+^lin^−^doublet^−^ Treg cells was determined by flow cytometry (FACS Canto II*™, *Becton Dickinson). After the isolation, 98.0% (median, range 97–99.5%) of the cells presented this phenotype*.

***(ii) Secreted molecules***

Molecules that are, or are not, secreted by the cells are useful to identify. These include cytokines (e.g., IL-10) and other soluble mediators. You **should** describe: (1) what you measured, (2) If using Abs, clone, conjugate and source of all antibodies and reagents used **must** be provided, (3) the methodology used for measurement, (4) cell density/milliliter of medium and plastic ware (e.g., 96 w round/flat bottom), (5) when supernatant was collected for cytokine concentration measurement, and (6) whether the cells received a stimulus and for how long before the measurement was carried out.

*EXAMPLE: IFN-*γ*; ELISA; supernatant after 24 h of unstimulated cell culture*.

***(iii) Epigenetic modifications***

Epigenetic modification relevant to the characteristics **should** be described if determined. Method of detection DNA demethylation **should** be clearly described.

*EXAMPLE: the mean percentage of demethylated TSDR of the foxp3 gene in the Treg population was 7% (Epiontis, Berlin, Germany)*.

***(iv) Specificity***

Polyclonal or antigen-specific, especially genetic modifications to manipulate specificity **should** be described. You **should** describe: (1) what is the specificity of the cells, (2) the methodology used to obtain the specificity, and (3) the methodology used to confirm the specificity. To describe the specificity of your cells, you should use CD names when available (e.g., use CD19 instead of the alternative name B4)—a full list of regularly updated CD numbers can be found on the website run by the HCDM8 (human cell differentiation molecules). Otherwise, you may use databases, e.g., http://hla.alleles.org, for HLA alleles, Uniprot9 for proteins and ChEBI10 for non-protein organic molecules describing the targets for your cells.

*EXAMPLE: HLA-A2-specific CAR (A2-CAR) Tregs were generated with lentiviral vectors encoding an HLA-A2-specific CAR by cloning and sequencing the heavy- and light-chain variable regions of the mAb and fusing the resulting scFv to portions of CD8, CD28, and CD3*ζ *in a second-generation CAR structure. Tetramers made from HLA-A2 were used to confirm the specificity of binding the cells to HLA-A2*.

**(e) Cell numbers**

***(i) Absolute cell number***

You **should** indicate the total number of cells present after extraction, and how they have been counted.

*EXAMPLE: 980* × *10^6^ cells as determined by Coulter counting*.

***(ii) Viability***

You **should** indicate the percentage of cells that are alive, and how this has been determined. The percentage of apoptotic cells should be stated if determined (indicate whether the starting material is fresh or frozen).

*EXAMPLE: 95% viability as determined by trypan blue exclusion. 5% of CD3^+^ T-cells had a phenotype indicating early apoptosis (7-AAD^−^, AnnexinV^+^) as measured by flow cytometry*.

**(2) Expansion/differentiation**

The section describes the protocol that has been used for expansion/differentiation of the isolated cells described in the previous section (Section 1). This process will hereafter be referred to as the expansion/differentiation process.

**(a) Pre-culture conditions**

The conditions under which the cells are kept after isolation but before starting the expansion/differentiation process (the fluid and type of container they are kept in, and at what temperature) **should** be described. The indication whether the starting material is fresh or thawed **must** be provided. You **should** also indicate the length of time between cell extraction and start of the expansion/differentiation process.

*EXAMPLE: isolated cells were placed in PBS with1% human serum albumin in a Falcon tube and kept at room temperature for up to 30* *min before starting the culture*.

**(b) Culture conditions**

The conditions under which the cells are kept during the expansion/differentiation process **should** be stated.

***(i) Cell number***

The number of cells used for the expansion/differentiation process **should** be stated, if different from numbers stated in Section 1e*i*.

*EXAMPLE: in total 5* × *10^6^ cells were put into culture*

***(ii) Cell concentration***

The concentration of cells in the medium at the start of and throughout the expansion/differentiation process **should** be stated as cells/milliliter.

*EXAMPLE: cells were put into culture at a concentration of 1* × *10^6^ cells/ml*

***(iii) Culture medium***

The medium in which the cells are grown **must** be described, including its source, and whether it has any additives (e.g., antibiotics, inactivated serum), excluding the stimuli that are described later. If you use more than one type of medium, or refresh the medium during the culture, then you **should** describe that here.

EXAMPLE: X-VIVO15 (Lonza) supplemented with5% human male type AB-serum (Sigma)

***(iv) Culture container***

The physical container in which the culture is carried out. This can include tissue culture plates, tissue culture bags or flasks. You **should** state the type of container, size and manufacturer. You **should** also indicate the total cell culture volume per container or well, as well as the total number of containers used.

EXAMPLE: 20 ml of medium in a 100 ml MACS Good Manufacturing Practice (GMP) Cell Differentiation bag (Miltenyi Biotec); 1 bag

***(v) Culture environment***

Describe the physical environment in which the cells are kept during the expansion/differentiation process. This **should** include the temperature and CO_2_ concentration. You **should** note whether medium has been pre-warmed. You **may** describe the equipment used to maintain the culture environment.

EXAMPLE: 37°C, 5% CO_2_; Medium was pre-warmed to 37°C; Sanyo CO_2_ incubator

**(c) Expansion/Differentiation protocol**

The protocol that is used to expand/differentiate the cells **should** be described. This **must** include the type and source of cytokine(s) or other agent(s) added into the medium, and at what time point and concentration **should** be included. You **should** also state the total length of the culture period as well as the rounds of stimulation, rounds of culture change, and the number of cell passages.

*EXAMPLE: rapamycin (final concentration of 100* *nM; Rapamune^®^, Pfizer) was added on day 0, 2, 5, 7, and 9. IL-2 (final concentration of 500 IU/ml; Proleukin^®^, Novartis) was added on day 2, 5, 7, and 9. Cells were harvested on day 12*.

**(d) Stimulus**

It **should** be stated whether the cells are expanded/differentiated polyclonally or in an antigen-specific manner or against an alloantigen. The protein(s), antibody(ies), accessory cells or other preparation(s) (e.g., antigen-presenting cells; APCs) with which the cells are stimulated **must** be named. You **must** describe the source of the preparation, concentration, and time point(s) at which it/they are added to the cell culture. Restimulation conditions, if any, should also be stated.

*EXAMPLE: cells were stimulated with CD3/CD28 MACS GMP ExpAct Treg Beads (Miltenyi Biotec) at a 4:1 bead:cell ratio. Cells were stimulated with CD40-activated allogeneic B cells (30 Gy-irradiated) at a ratio of 10 B cells per nTreg cell*.

**(e) Storage**

The conditions in which the cells are kept after completion of the expansion/differentiation process, but before being used in any subsequent experimental assay or treatment **should** be described. You **should** indicate the fluid and temperature in/at what the cells are being kept, as well as the length of time. You **should** indicate if cells are being frozen, and give details on the freezing and thawing procedures, including cell recovery and viability after thawing. You **should** also indicate if cells are taken out of their culture environment for any length of time during the expansion/differentiation process (e.g., if cells are frozen before completion of this process, with the aim to resume it at a later date).

*EXAMPLE: cells were kept in PBS 1% human serum albumin (Sigma) in a 50* *ml Falcon tube at room temperature for a maximum of 2 h; Cells were frozen in FCS/10% DMSO*.

**(3) Cells after expansion/differentiation**

This section describes the characteristics and state of the cells at the end of the expansion/differentiation process described in the previous section (Section 2).

**(a) Phenotype**

Characteristics of the cells at the end of their expansion/differentiation, including their specificity and purity (e.g., as% of target cells) **must** be described. Where only a proportion of cells in the population display a characteristic, you **should** indicate the percentage. You **should** report on the stability of the phenotype and how you determined this. It **should** be indicated if the phenotype of the cells post-expansion was determined using fresh viable cells, or rather after a freeze–thaw cycle in a batched analysis.

***(i) Cell surface and intracellular markers***

A number of phenotypic markers help to define the Treg cellular phenotype and specificity and are associated with distinct expression levels of surface and intracellular proteins. These markers are often characteristic of the transcriptional program of a cellular lineage and provide important information regarding the phenotypic stability and function of resulting cell products. You **should** describe: (1) what you measured, (2) the methodology used for measurement (including information on reagents; if using mAbs, information on clonotype, conjugate and manufacturer) **must** be provided, (3) whether the cells received a stimulus and for how long before the measurement was carried out, and (4) the method used to set marker or population positivity (e.g., fluorescence minus one method). You **should** use CD names when available (e.g., use CD127 instead of the alternative name IL-7Rα)—a full list of regularly updated CD numbers can be found on the website run by the HCDM (see footnote 8) (human cell differentiation molecules). Otherwise, you **may** use databases, e.g., http://hla.alleles.org, for HLA alleles, Uniprot (see footnote 9) for proteins and ChEBI (see footnote 10) for non-protein organic molecules.

*EXAMPLE: intracellular IFN-*γ *and IL-17 expression was measured by flow cytometry after 4* *h incubation with 20* *ng/ml PMA and 1* *µg/ml Ionomycin in the presence of 1* *µl/ml GolgiPlug™* using the BD Cytofix/Cytoperm™ buffer set.

***(ii) Secreted molecules***

Indicate molecules that are, or are not, secreted by the cells. These include cytokines (e.g., IL-10) and other soluble mediators. You **should** describe: (1) what you measured, (2) if using mAbs, clone, conjugate, and source of all antibodies and reagents used **must** be provided, (3) the methodology used for the measurement, (4) cell density/ml of medium and plastic ware (e.g., 96 w round/flat bottom), (5) when supernatant was collected for cytokine concentration measurement, and (6) whether the cells received a stimulus and for how long before the measurement was carried out.

*EXAMPLE: soluble IFN-*γ, *TNF-*α, *IL-17, and IL-10 were measured in the cell culture supernatant at a cell density of 1* × *10^6^ cells/ml by ELISA according to the manufacturers’ instruction*.

***(iii) Epigenetic modifications***

Epigenetic modification relevant to the characteristics **should** be described if determined. Method of detection DNA demethylation **should** be clearly described.

*EXAMPLE: the mean percentage of demethylated TSDR of the foxp3 gene in the Treg population was 97% (Epiontis, Berlin, Germany)*.

**(b) Functional assay**

You **should** describe any characteristic of the cells that has been measured by a functional assay (type of assays). This could either be the response of the cells to some stimulus or the behavior of other biological entities after exposure to the cells. There should be a clear indication of how the percentage of suppression was calculated (i.e., include formula). Whenever accessory cells such as responder cells are included in the assay, source and phenotype should be described. Behavior such as expression/production of molecules (described in Section 3a) does not need to be included.

*EXAMPLE: proliferation-based suppression assay using CFSE labeled autologous CD4^+^CD25^−^ responder cells; IFN-*γ *based suppression assay*

**(c) Cell numbers**

***(i) Absolute cell number***

You **must** indicate the total number of cells present at the end of the expansion/differentiation process, and how they have been counted and fold expansion **should** be included.

*EXAMPLE: cell numbers were microscopically determined using C-Chip disposable counting chambers from NanoEnTek and fold expansion to day 0 was calculated*.

***(ii) Viability***

You **must** indicate the percentage of cells that are alive and how this has been determined **should** be included.

EXAMPLE: 83% viability as determined by trypan blue exclusion

**(d) Dosing**

Whenever cells are transferred into an organism, details about dosing **must** be given. For clinical applications, information on the vehicle (solvent/medium) as well as intermediate components (trace amounts possible) **must** be given.

*EXAMPLE: a single dose of 1* × *10^7^ total nucleated cells per kilogram of body weight in 50* *ml 0.9% NaCl was transfused i.v*.

**(e) Quality control**

If the cells were produced for a clinical trial, you **must** describe release criteria and any methods used to determine sterility, specificity, purity, and quality of the product.

**(4) About the protocol**

In this section, we describe the general features about the protocol as a whole.

**(a) Regulatory authority**

Information about whether the protocol being used has been validated or quality-controlled to standards agreed to by an external regulatory authority **must** be stated. You **should** state the name of this authority. Also you **should** state whether the protocol follows GMP.

EXAMPLE: Medicines and Health Regulatory Authority

**(b) Purpose**

You **must** describe the overall purpose of the production of the cells.

*EXAMPLE: prevention of transplant rejection; Treatment of patients affected by Crohns’ disease*.

**(c) The relationship between the organism of origin of the cells and the target organism**

You **must** state if the cell product is autologous/allogeneic/xenogeneic/syngeneic to the recipient.

*EXAMPLE: patients receiving allogeneic kidney transplants and autologous Tregs. B6 mice receiving allogeneic (BALB/c xB6) heart transplants and syngeneic (B6) Tregs*.

**(d) Contact details**

You **must** provide the name and contact information of the corresponding author(s).

**(e) Citation**

You **should** add information that your paper was written in accordance with the Minimum Information for T Regulatory Cells reporting guidelines.

^1^http://flowcyt.sourceforge.net/miflowcyt/ ^2^http://fged.org/projects/miame/ ^3^http://miataproject.org ^4^https://doi.org/10.7717/peerj.2300 ^5^http://www.ncbi.nlm.nih.gov/taxonomy/ ^6^http://www.uberon.org ^7^http://fme.biostr.washington.edu/FME ^8^http://www.hcdm.org/ ^9^http://www.uniprot.org/ ^10^https://www.ebi.ac.uk/chebi/

## Appendix B

**Table:**

(MITREG) Checklist			

**Must**	**Should**	**May**	

			**(1) Cells at the start of procedure**
			**(a) Essential information about the donor**
			***(i) Species and strain***
			Species
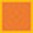
			Strain (if applicable)
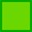
			***(ii) Characteristics of the organism***
			Health
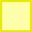
			Age
			Treatment/Environment
			Individual identifier number
			Source of purchase (if applicable)
			**(b) Source of cell material**
			Organ, tissue, fluid, or blood product
			Source (if applicable)
			Quantity (volume, size, or weight)
			Anti-coagulant (if applicable)
			If using cryopreserved sample
			Method and duration of storage
			Initial cell counts
			Ethical committee approval/written informed consent
			**(c) Cell separation process**
			***(i) Cell handling and labeling***
			Cell extraction method
			Tissue conditions between tissue retrieval and cell separation
			Duration
			Temperature
			Container
			Fluid
			Cell labeling
			Buffers and reagents (incl. source)
			Cell suspension volume and concentration
			Incubation temperature and duration
			Washing steps
			***(ii) Cell separation equipment and process***
			Methodology
			Equipment
			Presence of target cells in starting material described
			**(d) Phenotype**
			For any of the below, indicate the percentage of cells displaying the characteristic (if known)
			***(i) Cell surface and intracellular markers***
			Molecules measured [using cluster of differentiation (CD) names]
			Details of reagents used and source (incl. mAb clone, fluorochrome)
			Methodology
			Stimulus and time of stimulation (if applicable)
			Gating strategy to determine positive cells
			***(ii) Secreted molecules***
			Molecules measured
			Details of reagents used (incl. mAb clone, conjugate) and source
			Methodology
			Cell density/ml of medium and type of tissue culture plate
			Time point of supernatant collection
			Stimulus and time of stimulation (if applicable)
			***(iii) Epigenetic modifications***
			Epigenetic modification relevant to the characteristics
			***(iv) Specificity***
			Specificity of the cells (polyclonal or antigen-specific)
			Methodology used to obtain specificity
			Methodology used to confirm specificity
			**(e) Cell numbers**
			***(i) Absolute cell number***
			Total number of cells at the end of the isolation process
			Methodology
			***(ii) Viability***
			Percentage of viable cells
			Methodology
			**(2) Expansion/differentiation**
			**(a) Pre-culture conditions**
			Storage conditions
			Fluid
			Type of container
			Temperature
			Fresh or thawed
			Storage time
			**(b) Culture conditions**
			***(i) Cell number***
			The total number of cells put into culture
			***(ii) Cell concentration***
			The number of cells per ml of medium at start of culture
			***(iii) Culture medium***
			Type(s) of medium
			Source(s)
			Additives (excluding agents to maintain/induce T regulatory cells)
			Refreshment of the medium
			***(iv) Culture container***
			Type of container
			Size
			Manufacturer
			Cell culture volume per container or well
			Total number of containers or wells
			***(v) Culture environment***
			Temperature and CO_2_ concentration
			Use of pre-warmed medium
			Equipment
			**(c) Differentiation/tolerization protocol**
			Name of cytokine(s) or other agent(s) used
			Concentrations
			Time point(s) added to cell culture
			Total length of the culture period
			Rounds of stimulation
			Number of cell splitting
			**(d) Stimulus**
			Polyclonal/antigen-specific/alloantigen
			Stimulus (agent and/or accessory cell)
			Source
			Concentration
			Time point(s) added to culture
			Restimulation conditions (if applicable)
			**(e) Storage**
			Storage time
			Storage conditions
			If fresh
			Fluid
			Container
			Temperature
			If cryopreserved
			Freezing/thawing process
			Freezing medium
			Cell recovery and viability after thawing
			Time point at which cells are stored if different to the end of the culture process
			**(3) Cells after expansion/differentiation**
			**(a) Phenotype**
			For any of the below, indicate the percentage of cells displaying the characteristic (if known)
			Stability of the phenotype (if tested)
			Phenotype tested on fresh or thawed cells
			***(i) Cell surface and intracellular markers***
			Molecules measured (using CD names)
			Details of reagents used and source
			Methodology
			Stimulus and time of stimulation (if applicable)
			Gating strategy to determine positive cells
			***(ii) Secreted molecules***
			Molecules measured
			Details of reagents used and source
			Methodology
			Cell density/milliliter of medium and type of tissue culture plate
			Time point of supernatant collection
			Stimulus and time of stimulation (if applicable)
			***(iii) Epigenetic modifications***
			Epigenetic modification relevant to the characteristics
			**(b) Functional assay**
			Response of the cells to a defined stimulus
			Behaviour of other biological entities after exposure to the cells
			If using accessory cells, describe phenotype and source
			**(c) Cell numbers**
			***(i) Absolute cell number***
			Total number of cells at the end of the expansion process
			Methodology
			***(ii) Viability***
			Percentage of viable cells
			Methodology
			**(d) Dosing**
			Dose of cells transferred into organism (if applicable)
			Vehicle (solvent/medium) and intermediate components (for clinical trials only)
			**(e) Quality control (for clinical trial only)**
			Specificity
			Purity
			Sterility
			Potency
			**(4) About the protocol**
			**(a) Regulatory authority**
			External authority that approved the protocol
			Does protocol follow Good Manufacturing Practice?
			**(b) Purpose**
			The disorder for which the cell treatment has been manufactured
			**(c) Relationship between the source organism for the cells and the target organism**
			Allogeneic/autologous/xenogeneic/syngeneic
			**(d) Contact details**
			Name and contact information of the corresponding author(s)
			**(e) Citation**
			Acknowledge the MITREG reporting guidelines
